# The impact of the COVID-19 pandemic on chlamydia infection in South Korea: a comparison between the pre-pandemic and during-pandemic periods

**DOI:** 10.3389/fpubh.2023.1167321

**Published:** 2023-05-09

**Authors:** Achangwa Chiara, Sukhyun Ryu, Jae-Heon Jung, Se-Min Hwang

**Affiliations:** ^1^Department of Preventive Medicine, Konyang University College of Medicine, Daejeon, Republic of Korea; ^2^Department of Public Health and Welfare, The Graduate School, Konyang University, Daejeon, Republic of Korea; ^3^Konyang University Myunggok Medical Research Institute, Daejeon, Republic of Korea

**Keywords:** chlamydia, surveillance, sexually transmitted infection (STI), sexually transmitted disease (STD), SARS-CoV-2, Korea

## Abstract

**Background:**

Prior to COVID-19 pandemic, a yearly upward trajectory in the number of chlamydia infection cases was observed in South Korea. However, in response to the COVID-19 pandemic, Korea implemented several public health and social measures, which were shown to have an impact on the epidemiology of other infectious diseases. This study aimed to estimate the impact of the COVID-19 pandemic on the incidence and number of reported chlamydia infections in South Korea.

**Methods:**

Using the monthly number of reported chlamydia infection data between 2017 and 2022, we compared the trends in the reported numbers, and the incidence rates (IR) of chlamydia infection stratified by demographic characteristics (sex, age group, and region) in the pre- and during COVID-19 pandemic period (January 2017–December 2019 and January 2020–December 2022).

**Results:**

We observed an irregular downward trajectory in the number of chlamydia infection in the during-pandemic period. A 30% decrease in the total number of chlamydia infection was estimated in the during-pandemic compared to the pre-pandemic period, with the decrease greater among males (35%) than females (25%). In addition, there was a decrease in the cumulative incidence rate of the during COVID-19 pandemic period (IR: 0.43; 95% CI: 0.42–0.44) compared to the pre-pandemic period (IR: 0.60; 95% CI: 0.59–0.61).

**Conclusions:**

We identified decrease in the number of chlamydia infection during COVID-19 pandemic which is likely due to underdiagnosis and underreporting for the infection. Therefore, strengthening surveillance for sexually transmitted infections including chlamydia is warranted for an effective and timely response in case of an unexpected rebound in the number of the infections.

## Introduction

Chlamydia infection caused by *Chlamydia trachomatis* is a common sexually transmitted infection (STI) worldwide. Globally, ~50%−88% of chlamydia infection cases are asymptomatic (1). Due to the high rate of asymptomatic cases, systematic monitoring of chlamydia infections for high-risk populations, including sexually active young adults, has been recommended (2, 3). Furthermore, the monitoring of the infections provides us with a useful proxy for changes in sexual risk behaviors (4).

The first case of the newly discovered severe acute respiratory syndrome coronavirus 2 (SARS-CoV-2) in South Korea was reported on 20 January 2020 (5). And in an attempt to control the SARS-CoV-2, South Korea, implemented several public health and social measures (PHSMs), including social distancing, and wearing of face masks from 22 March 2020, which were crucial in mitigating the burden of the COVID-19 pandemic (6). The implementation of these PHSMs had considerable effects on the incidence and prevalence rates of other bacterial and viral infectious diseases (7, 8). In addition, these PHSMs reduced person-to-person interactions as well as the access and use of clinical services such as screening, and treatment for sexually transmitted infections (STIs) (9). The reported shift in various screening services including STI screening services, toward only symptomatic patients, could reduce the ability to identify asymptomatic chlamydia infections, hence a reduction in the overall reported number of cases (10).

There have been several concerns among clinicians and researchers in different countries regarding the spread of STIs during the COVID-19 pandemic and the possible long-term consequences of their underdiagnosis (11).

In 2019 before the importation of SARS-CoV-2 in South Korea, a total of 48,756 outpatient screening for chlamydia infection was done compared to 40,640 screenings in 2020 and 37,632 in 2021, implying a 16% and 23% decrease in 2020 and 2022, respectively (12).

Previous empirical literature in Europe (13, 14) and the United States (15) have demonstrated a decrease in the number of new cases of sexually transmitted infections including chlamydia infection during the early stages of the COVID-19 pandemic, and during the period when the PHSMs were implemented. In South Korea, a study reported an apparent decrease in the incidence of chlamydia infections during the early stages of the COVID-19 pandemic (16). However, the impact of COVID-19 on the trends of the reported number of chlamydia infection and incidence in the pre- vs. during-COVID-19 pandemic period in Korea is yet to be compared. Here, we compared the monthly number of reported chlamydia infection cases in the pre-COVID-19 period (January 2017–December 2019) with the number of monthly cases reported in the same period during-COVID-19 (January 2020–December 2022) by age, sex and regions in South Korea.

## Materials and methods

### Study data and variables

In South Korea, sentinel-based surveillance for chlamydia infection has been conducted since 2000 by the Korea Disease Control and Prevention Agency (KDCA) (17), an electronic database that collects data reported from healthcare professionals and laboratories in South Korea. This surveillance includes data from 587 sentinel sites located throughout the country (18). We collected the monthly number of chlamydia infection cases between January 2017 and December 2022, with a focus on the month of confirmation of the first COVID-19 case in South Korea (serving as the cut-off point of the pre vs. during-COVID-19 pandemic periods) and the first period of implementation of PHSMs (22 March, 2020 to 01 November, 2020). We divided the study period into two; the pre-pandemic (January 2017–December 2019) and the during-pandemic (January 2020–December 2022) periods.

In July 2017, due to system maintenance schedules according to the KCDA reports, no data inputs were made into the database. Therefore, we assumed the number of cases for the period of maintenance to be the average number of cases for June and August 2017. The collected data were stratified by sex, age group, and region of residence.

### Data analysis

We assessed the impact of the COVID-19 pandemic and the implementation of the PHSMs on the number of reported chlamydia infection cases using two approaches. First, we compared chlamydia infection reported case monthly and yearly trends for the pre-COVID-19 pandemic period compared with the during-COVID-19 pandemic period and for each subgroup (sex, age group, and region). Second, using the pre-pandemic values as the baseline, we estimated the percentage changes in the absolute number of cases during the pandemic period. We examined significant differences in the changes between the pre-pandemic and during-pandemic values using the Chi-squared test. Using the 2021 population of South Korea as the standard population, we estimated the cumulative incidence rates of chlamydia and 95% confidence intervals (CI) for the pre-pandemic and during-pandemic period per 1,000. Then, we conducted stratified analyses by age group in years (<20, 20–29, 30–39, 40–49, and ≥50), sex (male, and female), and region. We classified the regions into two categories; in the Seoul Capital Area (SCA) which was made up of Seoul, Incheon, and Gyeonggi; and out of SCA which included all other cities.

All analyses were performed using R version 4.2.0 (R Foundation for Statistical Computing, Vienna, Austria), and the level of statistical significance was set at *p* < 0.05.

## Results

A total of 31, 238 new chlamydia infection cases were reported in the pre-pandemic period while 22,186 new cases were reported in the during-pandemic period. There was a 28.9% decrease in the total number of chlamydia infection cases in the during-pandemic period compared to the pre-pandemic period. Stratifying by sex, compared to females (−24.9%), there was a significantly greater decrease in the number of chlamydia infection cases in males (−34.9%, *P* < 0.001). By age group, we identified a significant decrease in the number of reported cases among all groups, with the highest decrease in the age group 40–49 years (−41.9%, *P* < 0.001). By region, an almost equal magnitude of decrease was observed in the number of chlamydia infection cases in both the Seoul capital area region and out of the Seoul capital area region (respectively, −28.6% and −28.5 %, *P* < 0.001) (Table 1).

**Table 1:** Overall reported number of chlamydia infection cases during the pre-COVID-19 pandemic and during COVID-19 pandemic periods in South Korea.

**Demographic characteristics**	**Pre-COVID-19 pandemic**	**During COVID-19 pandemic**	**Change in absolute value**	**Percentage Change (%)**	^*^P-value
**No. of cases**	31,238	22,186	−9,052	−28.9	<0.001
**Sex**					
Male	12,582	8,181	−4,401	−34.9	<0.001
Female	18,656	14,005	−4,651	−24.9	<0.001
**Age groups**					
<20	2,259	1,808	−451	−19.9	0.004
20–29	15,080	11,702	−3,378	−22.4	0.0021
30–39	7,516	4,603	−2,913	−38.7	<0.001
40–49	3,847	2,232	−1,615	−41.9	<0.001
≥50	2,537	1,841	−696	−27.4	0.003
**Region**					
In SCA	18,430	13,159	−5,271	−28.6	<0.001
Out of SCA	12,628	9,027	−3,601	−28.5	<0.001

* p-values for the difference between the pre-pandemic and during-pandemic period for each demographic variable gotten by Chi-square test; SCA, seoul capital area.

**Figure 1:**
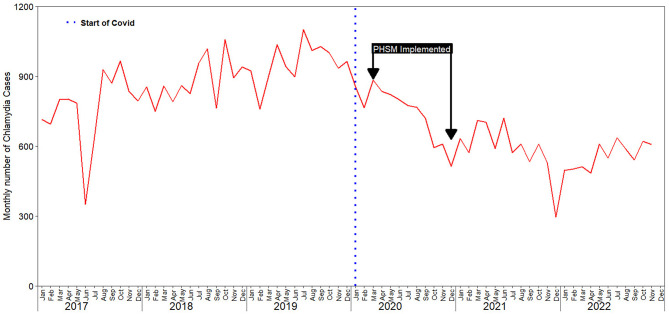
Impact of COVID-19 on chlamydia infection case reporting in South Korea: trends from 2017 to 2022.

We identified the monthly number of chlamydia infection cases reported had an irregular upward trajectory between 2017 and 2019, while, a downward trajectory was observed between 2020 and 2022 (Figure 1, Supplementary Figure S1).

**Figure 2:**
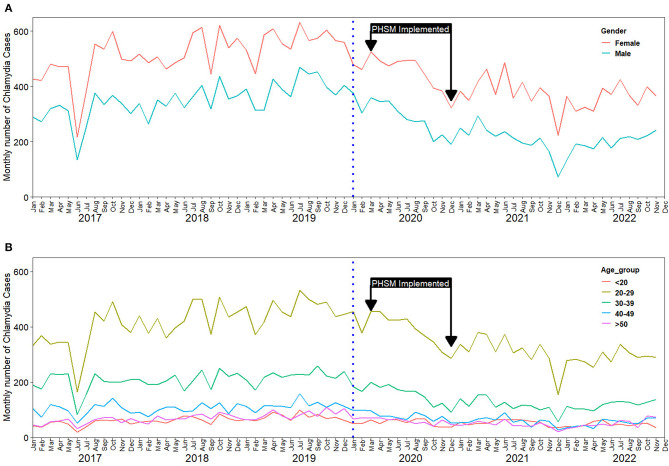
Impact of COVID-19 on chlamydia infection case reporting in South Korea: trends from 2017 to 2022; **(A)** Reported cases stratified by sex; **(B)** Reported cases stratified by age.

**Figure 3:**
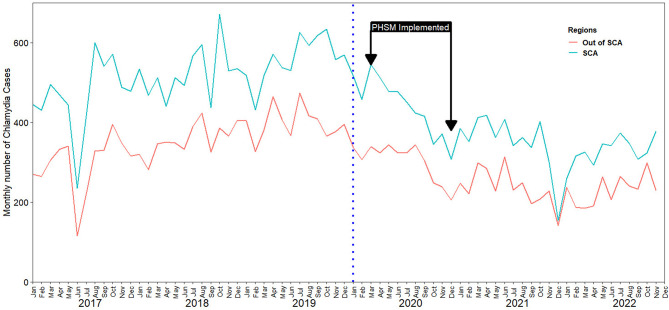
Impact of COVID-19 on chlamydia infection case reporting in South Korea by region.

Stratifying by sex, and age group an irregular downward trajectory was also observed in each subgroup, in the during-pandemic period (Figure 2). Similarly, we observed a downward trajectory in the during-pandemic period after stratification by region (Figure 3).

We also identified concurrent decreases in the during-pandemic chlamydia infection incidence rates in all subgroups in the during-pandemic period compared to the pre-pandemic period (Table 2).

**Table 2:** Changes in Chlamydia infection incidence rates (IR) per 1,000 population for the pre-COVID-19 pandemic and during COVID-19 pandemic periods in Korea.

		**Pre-COVID-19 pandemic period IR (95% CI)**								**During COVID-19 pandemic period IR (95% CI)**							
		**Overall**	**Sex**		**Age group (years)**					**Overall**	**Sex**		**Age group (years)**				
Overall		0.60 (0.59–0.61)	Male	Female	<20	20–29	30–39	40–49	≥50	0.43 (0.42–0.44)	Male	Female	<20	20–29	30–39	40–49	≥50
			0.49 (0.48–0.50)	0.72 (0.71–0.73)	0.27 (0.26–0.28)	2.34 (2.30–2.37)	1.08 (1.05–1.10)	0.47 (0.46–0.49)	0.12 (0.12–0.13)		0.32 (0.31–0.33)	0.54 (0.53–0.55)	0.21 (0.20–0.22)	1.81 (1.78–1.85)	0.66 (0.64–0.68)	0.27 (0.26–0.28)	0.09 (0.083–0.091)
Region	In SCA	0.71 (0.69–0.72)	0.55 (0.54–0.57)	0.88 (0.86–0.89)	0.31 (0.29 0.33)	2.61 (2.55–2.66)	1.17 (1.13–1.20)	0.51 (0.49–0.53)	0.13 (0.12–0.14)	0.51 (0.49–0.52)	0.37 (0.36–0.39)	0.63 (0.62–0.65)	0.24 (0.23–0.26)	1.98 (1.93–2.02)	0.73 (0.71–0.76)	0.29 (0.28–0.31)	0.10 (0.09–0.10)
	Out of SCA	0.49 (0.48–0.50)	0.43 (0.41–0.44)	0.56 (0.548–0.57)	0.22 (0.20–0.23)	1.99 (1.94–2.04)	0.97 (0.94–1.01)	0.43 (0.41–0.45)	0.11 (0.10–0.12)	0.35 (0.35 0.36)	0.26 (0.25–0.27)	0.45 (0.43–0.46)	0.18 (0.17–0.20)	1.60 (1.56–1.65)	0.57 (0.55–0.59)	0.25 (0.24–0.27)	0.08 (0.07–0.08)

SCA, seoul capital area.

## Discussion

We assessed the impact of the COVID-19 pandemic on the number of reported chlamydia infection cases and the incidence of chlamydia infection in Korea using national surveillance data. This assessment demonstrates the substantial impact of the COVID-19 pandemic on the chlamydia prevention program.

We observed a continuous downward trajectory in the number of reported chlamydia infection cases across all the years in the during-pandemic period. Similarly, there was a decrease in the cumulative incidence rate in the during-pandemic period compared to the pre-pandemic period. A similar trend was observed with other STIs (Supplementary material). This observed decreases in the pandemic period could be a result of behavioral changes as part of the stay-at-home mandates and also as a result of some barriers to patient care and preventive services that were directly or indirectly introduced as a result of the COVID-19 pandemic. During the COVID-19 pandemic, individuals were very reluctant to visit the doctor's office for medical consultation for fear of SARS-CoV-2 infection (19, 20). These changes in health-seeking behaviors could have impacted the screening of asymptomatic individuals. Moreover, the constant surveillance of STIs done by the local public health centers as well as associated primary health clinics may have been interrupted by COVID-19 control activities, limiting doctor's appointments and screening of potential cases. Our findings are similar to other studies that have also reported a decrease in the incidence and number of reported cases of chlamydia infection and other STIs in the pandemic period compared to the pre-pandemic period (21–24). However, our results are contrary to a study that reported an increase in chlamydia infection cases and other STIs during the COVID-19 pandemic (25, 26).

In this study, a sharper decrease in the overall trend was observed after the implementation of the PHSMs. During this period, the activities of the adult entertainment sectors (including nightclubs, bars, and other nighttime activities) were shut down. This may have reduced the number of STI screenings, as workers in the adult entertainment sector in South Korea are mandatorily screened for STIs every 3 months according to Korean Infectious Disease Control and Prevention act. However, due to the limited public health resources during COVID-19 pandemic, many workers was not likely screened for STIs by public health authorities. Our results are contrary to previous research that showed that PHSMs implemented during the early pandemic did not affect STIs (15, 27). This is likely due to the different level of use for public health resources for COVID-19 pandemic in different countries.

Our study also showed a sex, age group, and regional decrease in the trend of incidence and number of reported cases of chlamydia infection in the during-pandemic compared to the pre-pandemic period. Specifically, a significantly greater decrease was observed in males (−34.9%) compared to females and among those between 40 and 49 years old (−41.9%) compared to other age groups. This is in line with other studies in the literature which have shown that males are less likely to seek health care, especially for preventive care visits (28, 29). Therefore, this could be explained by underdiagnosis and underreporting given that there are concerns that chlamydia infections may be underdiagnosed in males and in middle age individuals (30). Underdiagnosis and underreporting of chlamydia infections may be due to decreased screening during the pandemic. Appropriate screening and medical consultations are recommended and health education and promotion activities aimed at sensitizing the public and healthcare providers are also needed.

There are some limitations to the present study. First, this study does not take into account the number of chlamydia tests that were conducted. Although the number of reported chlamydia infection cases decreased in the during-pandemic period, it is highly likely that many screening tests for the infection were not conducted. Furthermore, it is possible that the decrease in reported cases was not only due to the COVID-19 pandemic, but potentially due to policy changes around the fear of exposure to SARS-CoV-2 infection in a clinical setting.

Secondly, although several cofactors may exist between the COVID-19 pandemic and the incidence and number of reported chlamydia cases, this study could not assess the magnitude of the effect of each mediator, such as social restrictions, physical distancing, and hygiene measures.

In conclusion, our results suggest that the incidence and number of chlamydia infection cases decreased during the COVID-19 pandemic in South Korea. In pandemic and epidemic emergencies that involve behavioral restrictions, the promotion of healthcare-seeking behaviors among high-risk individuals for the sexually transmitted infections including chlamydia is encouraged.

## Data availability statement

Publicly available datasets were analyzed in this study. This data can be found here: https://www.kdca.go.kr/npt/biz/npp/ist/simple/simplePdStatsMain.do.

## Ethics statement

Ethical review or informed consent was not required because all data used in this study were anonymous and publicly available.

## Author contributions

S-MH and SR conceived the study and sought funding. SR, AC, and J-HJ did the data collection, assimilation, and data analysis. SR and AC wrote the first draft of the manuscript. S-MH and AC critically reviewed and edited the manuscript. All authors contributed to the interpretation of the results, critical revision of the manuscript, and have given final approval of the version to be published.
