# Supplementary material for: The impact of the COVID-19 pandemic on chlamydia infection in South Korea: a comparison between the pre-pandemic and during-pandemic periods
Source: Front Public Health. 2023 May 9;11:1167321. doi: 10.3389/fpubh.2023.1167321 (PMC10203704; doi:10.3389/fpubh.2023.1167321)
Supplement: Supplementary file 1 [file Table_1.docx]

# Supplementary

**The impact of the COVID19 pandemic on Chlamydia infection in South Korea: A comparison between the pre-pandemic and during-pandemic periods.**

# Additional information

We collected additional data on the number of cases for the other sexually transmitted infections (STIs) including; primary, secondary, and congenital Syphilis, Gonorrhea, and Chancroid reported by the KDCA and estimated the pre-pandemic and during-pandemic changes.

Similar to chlamydia, compared to the pre-pandemic period, significant decreases in the overall number of cases for other STIs in the pandemic period (primary Syphilis (-84.77%), secondary Syphilis (-80.24%) congenital Syphilis (-79.03%), Gonorrhea (-26.01%), and Chancroid (-81.82%) were observed (Supplementary Table 1).

**Supplementary Table 1**: Overall reported number of sexually transmitted infections including primary, secondary, congenital syphilis, gonorrhea, and chancroid, cases during the pre-COVID-19 pandemic and during the COVID-19 pandemic in South Korea.

| **Characteristics** | **Pre-COVID-19 era** | **During- COVID-19 era** | **Change in absolute value** | **Percentage Change (%)** | **P-values** |
| --- | --- | --- | --- | --- | --- |
| **Primary syphilis (***Treponema pallidum***)** | | | | | |
| Total number of cases | 4,201 | 640 | -3,561 | -84.77 | <0.001 |
| Sex | | | | | |
| Male | 3,019 | 436 | -2,583 | -85.56 | <0.001 |
| Female | 1,182 | 204 | -978 | -82.74 | <0.001 |
| Age groups | | | | | |
| < 20 | 220 | 41 | -179 | -81.36 | <0.001 |
| 20-29 | 1,463 | 212 | -1,251 | -85.51 | <0.001 |
| 30-39 | 1,014 | 161 | -853 | -84.12 | <0.001 |
| 40-49 | 625 | 92 | -533 | -85.28 | <0.001 |
| ≥ 50 | 879 | 138 | -741 | -84.30 | <0.001 |
| Region | | | | | |
| In SCA | 2,401 | 400 | -2,001 | -83.34 | <0.001 |
| Out of SCA | 1,800 | 240 | -1,560 | -86.67 | <0.001 |
| **Secondary syphilis** | | | | | |
| Total number of cases | 1,918 | 379 | -1,539 | **-**80.24 | <0.001 |
| Sex | | | | | |
| Male | 1,381 | 276 | -1,105 | -80.01 | <0.001 |
| Female | 537 | 103 | -434 | -80.82 | <0.001 |
| Age groups | | | | | |
| < 20 | 109 | 10 | -99 | -90.83 | <0.001 |
| 20-29 | 749 | 175 | -574 | -76.64 |  |
| 30-39 | 340 | 103 | -237 | -69.71 |  |
| 40-49 | 242 | 40 | -202 | -83.47 | <0.001 |
| ≥ 50 | 408 | 52 | -356 | -87.25 | <0.001 |
| Region | | | | | |
| In SCA | 1,150 | 301 | -849 | -73.83 |  |
| Out of SCA | 768 | 78 | -690 | -89.84 | <0.001 |
| **Congenital syphilis** | | | | | |
| Total number of cases | 62 | 13 | -49 | **-**79.03 | 0.002 |
| Sex | | | | | |
| Male | 31 | 7 | -24 | -77.42 | 0.002 |
| Female | 31 | 6 | -25 | -80.65 | <0.001 |
| Age groups | | | | | |
| < 20 | 62 | 13 | -49 | **-**79.03 | 0.002 |
| 20-29 | 0 | 0 | 0 | 0 | 0 |
| 30-39 | 0 | 0 | 0 | 0 | 0 |
| 40-49 | 0 | 0 | 0 | 0 | 0 |
| ≥ 50 | 0 | 0 | 0 | 0 | 0 |
| Region | | | | | |
| In SCA | 22 | 8 | -14 | -63.64 | 0.0023 |
| Out of SCA | 40 | 5 | -35 | -87.50 | <0.001 |
| **Gonorrhea** (*Neisseria gonorrhoeae*) | | | | | |
| Total number of cases | 7,124 | 5,271 | -1,853 | -26.01 | <0.001 |
| Sex | | | | | |
| Male | 5,010 | 3,534 | -1,476 | -29.46 | <0.001 |
| Female | 2,114 | 1,737 | -377 | -17.83 | 0.003 |
| Age groups | | | | | |
| < 20 | 714 | 516 | -198 | -27.73 | <0.001 |
| 20-29 | 2,924 | 2,330 | -594 | -20.31 | 0.002 |
| 30-39 | 1,798 | 1,237 | -561 | -31.20 | <0.001 |
| 40-49 | 1,050 | 693 | -357 | -34.00 | <0.001 |
| ≥ 50 | 638 | 495 | -143 | -22.41 | 0.0021 |
| Region | | | | | |
| In SCA | 4,410 | 3,189 | -1,221 | -27.69 | <0.001 |
| Out of SCA | 2,714 | 2,082 | -632 | -23.29 | 0.0025 |
| **Chancroid** (*Haemophilus ducreyi*) | | | | | |
| Total number of cases | 11 | 2 | -9 | -81.82 | 0.631 |
| Sex | | | | | |
| Male | 4 | 1 | -3 | -75.00 | 0.67 |
| Female | 7 | 1 | -6 | -85.71 | 0.65 |
| Age groups | | | | | |
| < 20 | 2 | 0 | -2 | -100 | 0.81 |
| 20-29 | 5 | 0 | -5 | -100 | 0.91 |
| 30-39 | 2 | 0 | -2 | -100 | 0.81 |
| 40-49 | 1 | 0 | -1 | -100 | 0.93 |
| ≥ 50 | 1 | 2 | 1 | 100 | 0.79 |
| Region | | | | | |
| In SCA | 6 | 1 | -5 | -83.33 | 0.61 |
| Out of SCA | 5 | 1 | -4 | -80.00 | 0.53 |

*p-values for the difference between the pre-pandemic and during-pandemic period for each demographic variable gotten by Chi-square test; SCA (Seoul Capital Area)

**Supplementary Figure 1 (S1):** Yearly reported number of chlamydia infection cases
