# Supplementary figures and images for: The impact of the COVID-19 pandemic on chlamydia infection in South Korea: a comparison between the pre-pandemic and during-pandemic periods
Source: Front Public Health. 2023 May 9;11:1167321. doi: 10.3389/fpubh.2023.1167321 (PMC10203704; doi:10.3389/fpubh.2023.1167321)

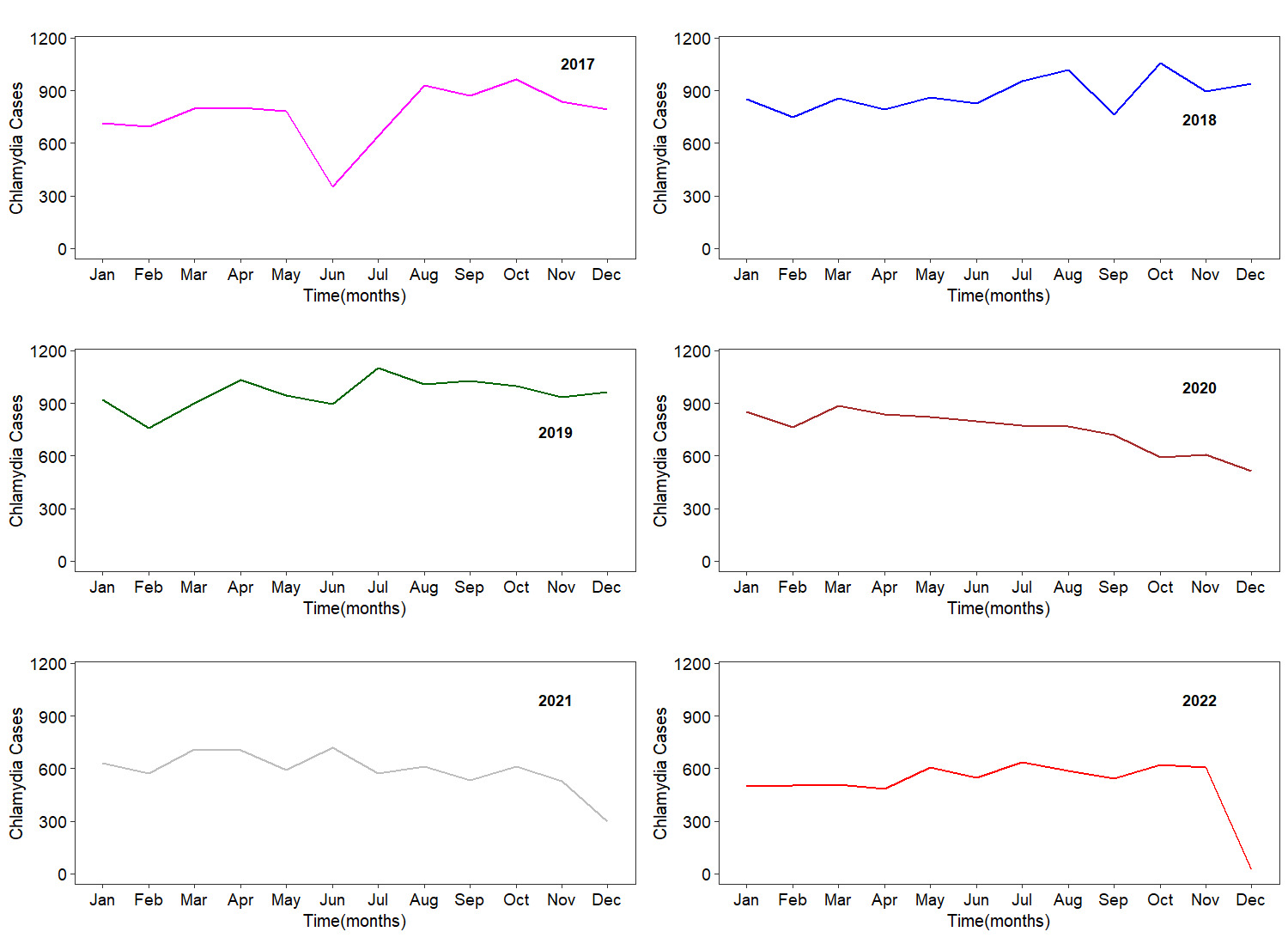

Supplement: Supplementary file 2 [file Image_1.JPEG]
